# Cancer immunenet biomarker profiling panel

**DOI:** 10.1186/2051-1426-3-S2-P82

**Published:** 2015-11-04

**Authors:** Alex Chenchik, Mikhail Makhanov, Leonid Iakoubov, Costa Frangou

**Affiliations:** 1 Cellecta, Inc., Mountain View, CA, USA

## Background

Several recent studies have now firmly established a link between the immune infiltrate in several human carcinoma types and prognosis and/or response to therapy. Correspondingly, increasing evidence suggests that the number, type, and location of tumor-infiltrating lymphocytes in primary tumors harbor prognostic value, and this has led to the development of a “tumor immunoscore/immune index.”

## Methods

The aim of this study was to develop Driver-Map™ ImmuneNet, a comprehensive cancer immuno-panel of over one thousand genes to quantitatively assess the transcriptome profile of the tumor microenvironment (e.g. infiltrating immune cells, blood vessels, and stroma cells) and allow accurate predictions of tumor purity. This ImmuneNet panel includes approximately 400 immunity-related genes from 16 predictive and prognostic core gene signatures that have been validated in recent chemo- and immunotherapy clinical trials across several tumor types, including melanoma, colorectal, breast, and lung cancers. In order to expand the core gene signatures, we developed a non-probabilistic binary linear classifier algorithm to infer the level of infiltrating immune cells in tumor tissues using a novel multiplex Driver-Map pipeline. The ImmuneNet panel also includes a comprehensive set of genes specific for detection and quantitative profiling of different types of activated immune cells of adaptive and innate immunity, fibroblasts, stromal and endothelial cells in the tumor microenvironment.

## Results

Accordingly, the ImmuneNet panel allows quantitative profiling of the expression levels of over 1000 key cancer immuno-related genes using multiplex RT-PCR amplification from 10-100ng of total RNA that is followed by Next-Gen Sequencing. The built-in internal calibration standards for each target gene allows calibration and adjusting of digital NGS data depending on the level of intrinsic noise and quality of samples. The ImmuneNet panel is designed to be a robust platform with superior sensitivity suitable for a variety of clinical samples. To demonstrate the performance and utility of this panel, we will present profiling data from infiltrating immune cells and key intact and deficient immune mechanisms in the tumor microenvironment of breast cancer samples. Comprehensive profiling of tumor-associated immune cells with this gene panel will enable researchers to discover prognostic and predictive immune response biomarkers, to stratify cancer patients for responses to the growing number of immunotherapeutic treatments.

## Conclusions

ImmuneNet will not only enable large-scale analysis of complex RNA mixtures for the discovery of novel cancer biomarkers and therapeutic targets, it can be a useful platform for exploring infiltrating cell types and obtaining information of immune status.

